# Supplementary material for: Interaction kinetics between p115-RhoGEF and Gα13 are determined by unique molecular interactions affecting agonist sensitivity
Source: Commun Biol. 2022 Nov 24;5:1287. doi: 10.1038/s42003-022-04224-9 (PMC9700851; doi:10.1038/s42003-022-04224-9)
Supplement: Supplementary file 2 — Supplementary Information [file 42003_2022_4224_MOESM2_ESM.pdf]

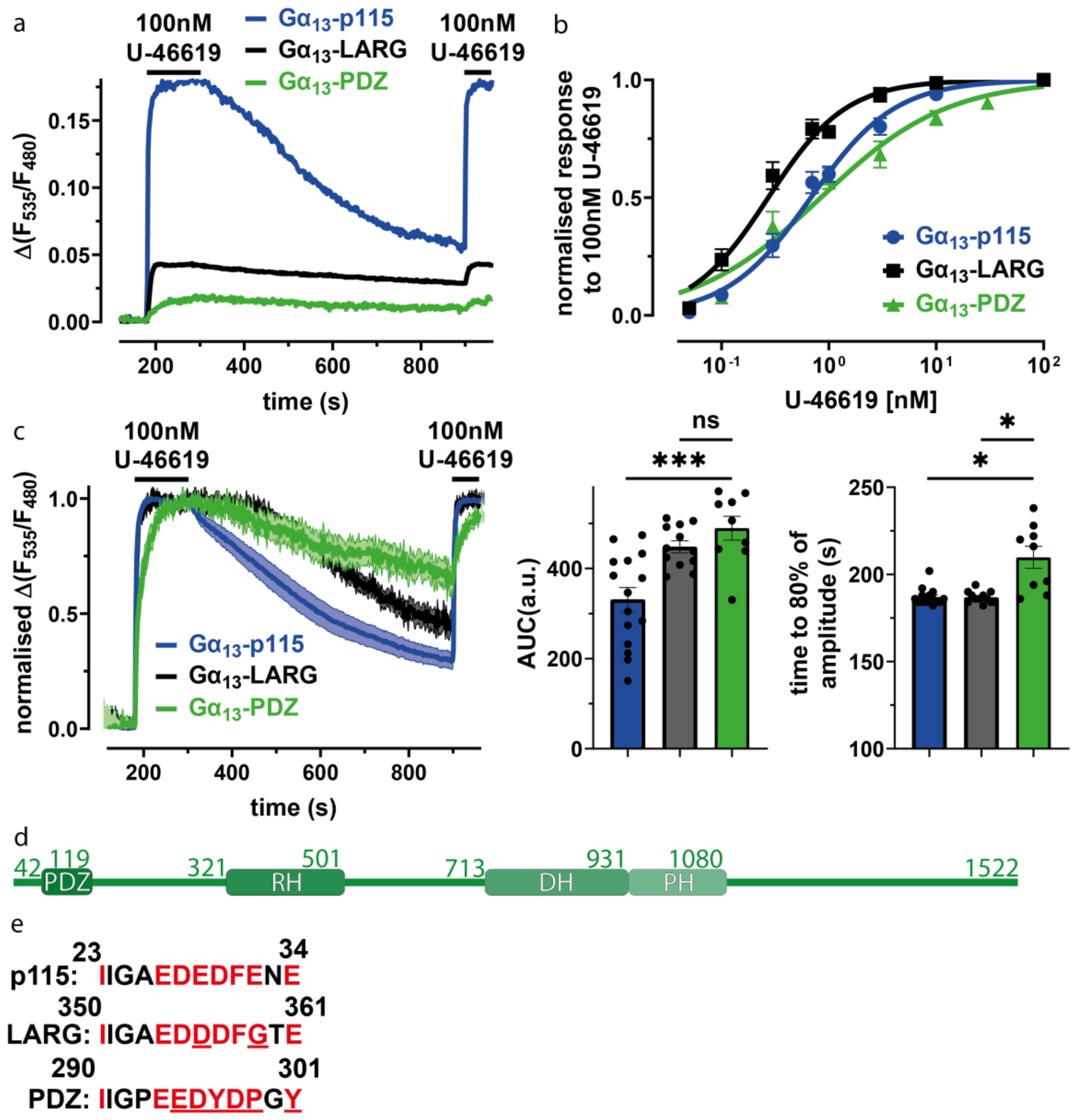

Supplementary Fig. 1 Classification of the Gα$_{13}$-PDZ-RhoGEF interaction a) Representative traces of the interaction of Gα$_{13}$ with either p115 [blue], LARG [black], or PDZ-RhoGEF [green] (values only corrected, but not normalized). With 0.18 the amplitude of the Gα$_{13}$-p115 interaction is about 4.2 times higher than the amplitude of the Gα$_{13}$-LARG (0.043) interaction and ten times higher than the amplitude of the Gα$_{13}$-PDZ-RhoGEF interaction (0.018). The amplitude of the Gα$_{13}$-LARG interaction is about 2.4 times higher than the amplitude of the Gα$_{13}$-PDZ interaction. **b)** Comparison of the Gα$_{13}$-PDZ-RhoGEF [green] concentration-response curve with p115-RhoGEF and LARG (data acquisition and evaluation described in Fig. **2a**/ Fig. **2b**; data for p115-RhoGEF [blue] and LARG [black] taken from Fig. **2b**). With an EC_{50} value of 0.84 nM the Gα$_{13}$-PDZ-RhoGEF interaction shows a concentration-response curve, that is similar to the concentration-response curve of the Gα$_{13}$-p115 interaction, although less steep, with the only significant [**P=0.0087, unpaired t-test] difference in sensitivity at 10 nM U-46619. **c)** The Gα$_{13}$-PDZ-RhoGEF interaction [green, n=9, mean AUC= 489.3] displayed slow off kinetics: the AUC of decay (described in Fig. **2c**) is significantly [***P=0.0008] larger than the AUC of the Gα$_{13}$-p115 interaction [blue, data taken from Fig. **2c**], while no significant difference was found compared to Gα$_{13}$-LARG [black, data taken from Fig. **2c,** Brown-Forsythe and Welch ANOVA test with Dunnett´s T3 multiple comparison test, $^{ns}P=0.3013$]. In addition, the on-kinetics of the Gα$_{13}$-PDZ-RhoGEF interaction was slower than for the other RH-RhoGEFs as the time required to reach 80% of the maximum response was significantly higher [Brown-Forsythe and Welch ANOVA test with Dunnett´s T3 multiple comparison test; *P=0.0121, *P=0.0119]. **d)** Like LARG (see Fig. 3a), PDZ-RhoGEF contains the principal binding site for Gα$_{13}$: the RH domain, while also containing a DH/PH domain for effector binding, as well as a PDZ domain. **e)** Comparing the binding motif N-terminal of the RH domain that proved to be important for the Gα$_{13}$-p115 interaction: like LARG, PDZ-RhoGEF does not possess an acidic amino acid (P299) at the relevant position that would be necessary to interact with R128 of Gα$_{13}$. In addition many of the other amino acids that interact with Gα$_{13}$ (marked in red) deviate from the other RH-RhoGEFs sequence.

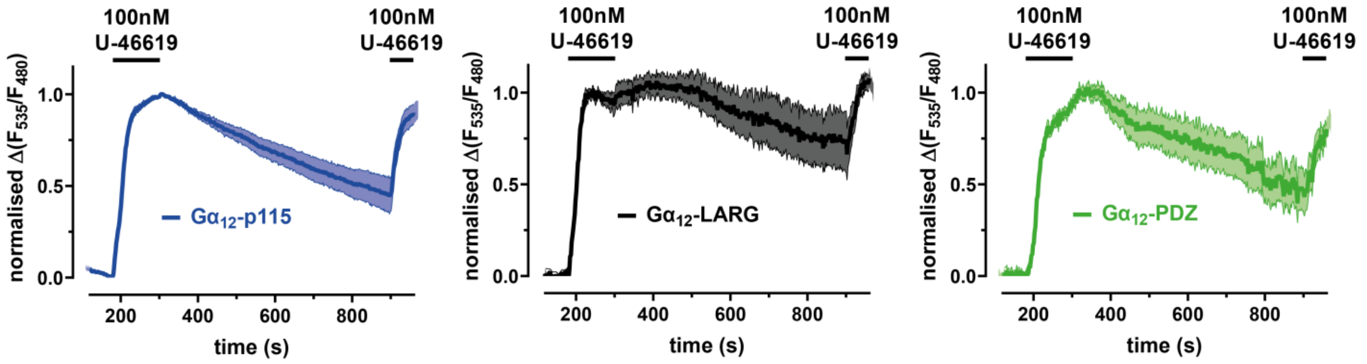

Supplementary Fig. 2 Gα$_{12}$-RH-RhoGEF interaction The second G protein that belongs to the Gα$_{12/13}$ family Gα$_{12}$ also showed agonist induced interaction in the described FRET based single cell assay.

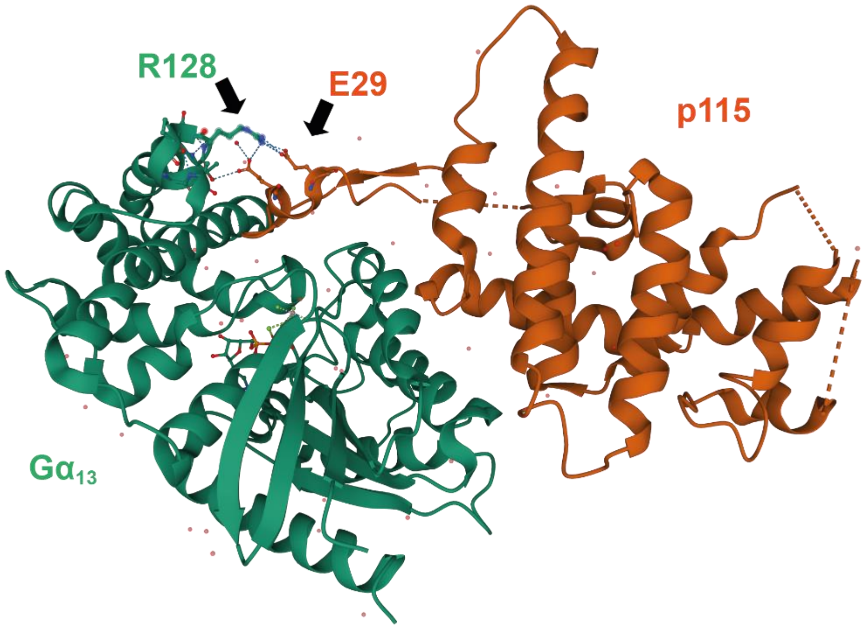

Supplementary Fig. 3 Overview of the Gα$_{13}$-p115 interaction The Gα$_{13}$ R128-p115 E29 interaction shown in Fig **5a** zoomed out for a better insight into the structural arrangement.

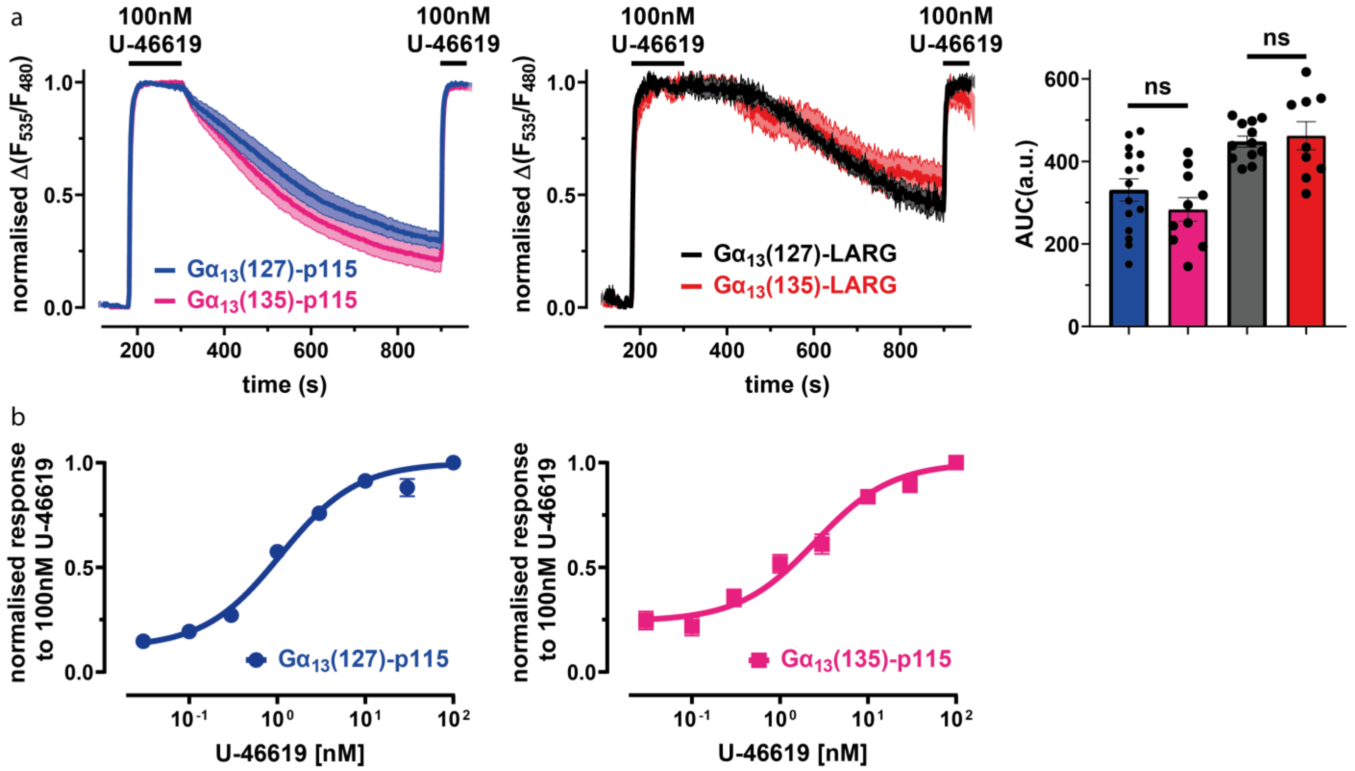

Supplementary Fig. 4 Interaction with Gα$_{13}$ variants a) In the FRET based Gα$_{13}$-RhoGEF interaction assay neither the Gα$_{13}$(135)-p115 [pink, n=10, mean AUC=283.6, unpaired t-test, ^{ns}P=0.2505], nor the Gα$_{13}$(135)-LARG interaction [red, n=9, mean AUC=462.2, unpaired t-test with Welch´s correction, ^{ns}P=0.7092] showed a significant difference in the AUC of decay compared to the interactions of their Gα$_{13}$(127) counterparts (data taken from Fig. **2c**). **b)** Additional concentration-response curves of the Gα$_{13}$(127)-p115 interaction [blue, measured in three triplets=nine wells per concentration; EC$_{50}$=1.09 nM] and the Gα$_{13}$(135)-p115 interaction [pink, three triplets; EC$_{50}$=2.52 nM] have been measured utilizing BRET in a 96-well plate format in a plate reader. Gα$_{13}$ was tagged with NLuc instead of mTurq2.

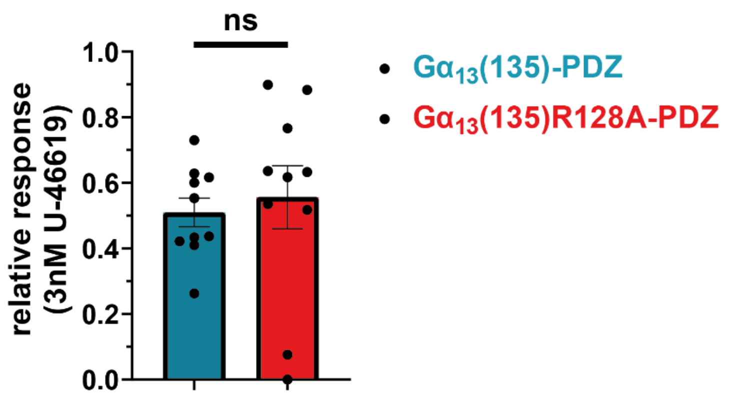

Supplementary Fig. 5 Comparison of the PDZ-RhoGEF interaction with Gα$_{13}$(135) and Gα$_{13}$(135)R128A No significant difference was found comparing the Gα$_{13}$(135)-PDZ-RhoGEF interaction [cyan, n=10, mean relative response=0.51] with the Gα$_{13}$(135)R128A-PDZ-RhoGEF interaction [red, n=10, mean relative response=0.56, Welch´s t-test; ^{ns}P=0.6632] in a FRET assay, where the response to 0,3 nM U-46619 was normalised to the maximum response of 100 nM U-46619 (for more information about Gα$_{13}$(135) and the Gα$_{13}$(135)R128A mutant see Methods and Figure **5d**).

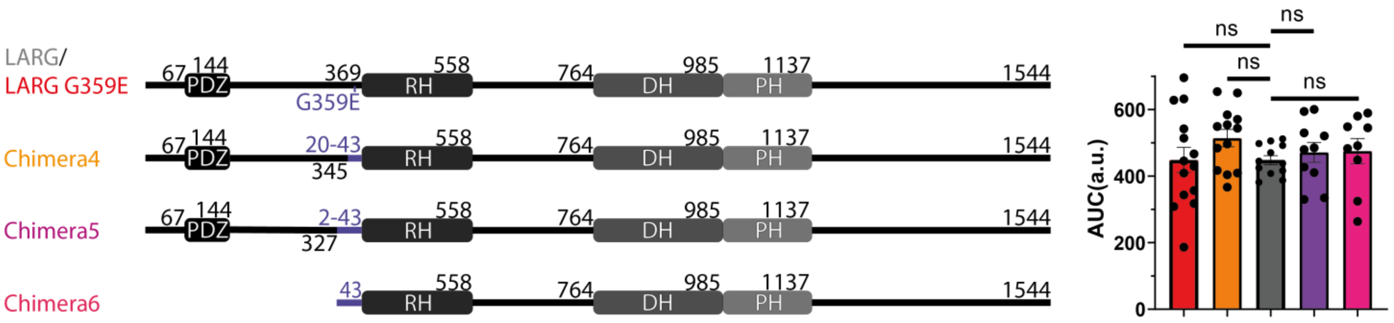

Supplementary Fig. 6 Insertion of the functional Gα$_{13}$ interaction motif of p115 into LARG failed to accelerate Gα$_{13}$-LARG dissociation The following mutants and chimeras did not show a significant difference in interaction time compared to Gα$_{13}$-LARG [grey, n=10 , mean AUC=448.1]: LARG G359E, a mutant where glycine on position 359 was modified to Glutamic acid [red, n=14, mean AUC=448.2; ^{ns}P=0.9999], Chimera4, where the p115 binding motif (amino acids 20-43) replaces the respective amino acids N-terminal of the RH-Domain of LARG [amino acids 346-369; orange, n=13 , mean AUC=514.1, ^{ns}P=0.1141], Chimera5 had the whole part N-terminal of p115 (amino acids 2-43 replaces LARG 328-369) inserted into full length LARG, with the rest of the N-terminal part of LARG still attached [purple, n=10, mean AUC=471.2, ^{ns}P=0.8924] and Chimera6, which was constructed like Chimera5, only with the N-terminus of LARG removed [p115 amino acids 1-43 instead of LARG 1- 369; pink, n=9, mean AUC=474.9, ^{ns}P=0.9049, Brown-Forsythe and Welch ANOVA test with Dunnett´s T3 multiple comparison test].

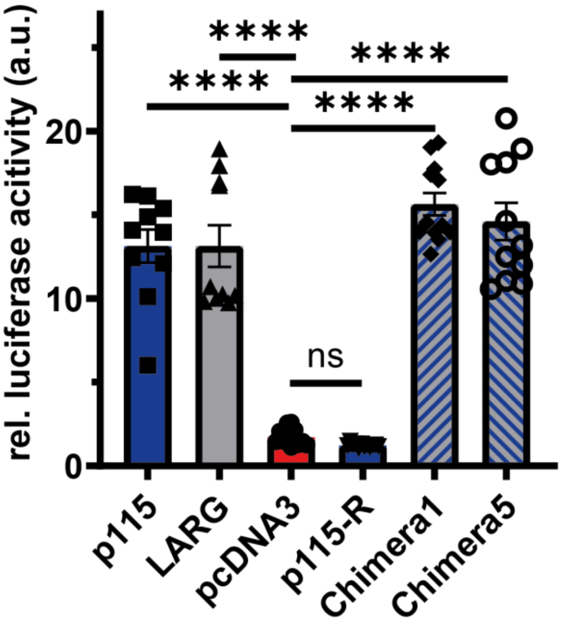

Supplementary Fig. 7 Dual-Luciferase Serum Response Factor Reporter Assay Cells transfected with p115-RhoGEF [blue, n=10, mean value: 13.14; ****P=0.0001], LARG [grey, n=10, mean value: 13.14; ****P=0.0001], Chimera1 [blue-grey, n=12, mean value: 15.64; ****P=0.0001], or Chimera5 [grey-blue, n=11, mean value: 14.61; ****P=0.0001] all showed significantly higher relative luciferase activity than cells transfected with an empty pcDNA3 vector [red, n=12, mean value: 1.72] in the Dual-Luciferase Reporter Assay System, while cells transfected with the truncated p115-R [blue, n=12, mean value: 1.23; ^{ns}P=0.9974; Ordinary one-way ANOVA with multiple comparisons] did not show a significant difference.

Gibson Assembly

Plasmid	Vector-Primer_forward 5´-3´	Vector-Primer_reverse 5´-3´	Fragment-Primer_forward 5´-3´	Fragment-Primer_reverse 5´-3´	Template Vector	Template Fragment
LARG-PR	gggagtaaaatgactcgagcatgcatctagagg	actgtgtgccactcatggatcccttgtacagctcgtccatgcc	atgagtggcacacagtctactatcac	gctcgagtcattttactcccaaatgcttcatatacatgagaataaca	p115	LARG
Chimera1	tggggtgcgggtgaaagagcctcgaaatttggagc	gcccctcgggcgaagtcttccatggatcccttgtacagctcg	atggaagacttcgcccgag	gctctttcacccgcaccccaaggtgg	LARG	p115
Chimera2	gggagtaaaaaccaagagtggagacaagaagtcg	ggaaacagctgttttgctcttctgagtttgtctc	agagcaaaacagctgtttccagagcattgaattactaaaat	cactcttggtttttactcccaaatgcttcatatacatgagaataaca	p115	LARG
Chimera3	ggtgcgggtgaaagagcctcgaaatttggagca	tggaactggctgcactgtccattgatctgtt	atggacagtgcagccagttccagagcctg	gaggctctttcacccgcaccccaaggtgg	LARG	p115-R
$G\alpha_{13}$(135)	acgagctgtacaaggctagcatggtggagactcgagtgttcc	cccttgctcaccatgctagccccctgggcagccatg	gctagcatggtgagcaaggg	gctagccttgtacagctcgtc	$G\alpha_{13}$(127)	$G\alpha_{13}$-wt
Chimera4	agagcaaaacagctgtttccagagcattgaattactaaaa	tgatgctgactgctatacgggttgagggacttc	ccgtatagcagtcagcatcatcggggctga	ctggaaacagctgttttgctcttctgagtttgtctc	LARG	p115
Chimera5	agagcaaaacagctgtttccagagcattgaattactaaaa	gccgcccctcgggcgaagtcttcacttttctctgggttttcctctagataaatt	gaagacttcgcccgaggg	ctggaaacagctgttttgctcttctgagtttgtctc	LARG	p115
Chimera6	agagcaaaacagctgtttccagagcattgaattactaaaa	gcccctcgggcgaagtcttccatggatcccttgtacagctcg	atggaagacttcgcccgag	ctggaaacagctgttttgctcttctgagtttgtctc	LARG	p115

Mutagenesis

Plasmid	Mutagenesis-Primer 5´-3´	Template
p115-E32G	ggatgaggattttgggaacgagctggagac	p115
$G\alpha_{13}$(135)R128A	gatggcatttgataccgccgcccccatggctgc	$G\alpha_{13}$(135)
LARG-G359E	gaagatgatgattttgaaactgaacatgaacag	LARG

Truncation by restriction enzymes

Plasmid	Restriction enzyme 1	Restriction enzyme 2	Template
p115-R	BamHI	XhoI	p115
p115-Rshort	AgeI	XhoI	p115
LARG-Rshort	BamHI	XhoI	LARG

Supplementary Table 1 List of primers and templates used for cloning and mutagenesis
