# Supplementary material for: Interaction kinetics between p115-RhoGEF and Gα13 are determined by unique molecular interactions affecting agonist sensitivity
Source: Commun Biol. 2022 Nov 24;5:1287. doi: 10.1038/s42003-022-04224-9 (PMC9700851; doi:10.1038/s42003-022-04224-9)
Supplement: Supplementary file 3 — Description of Additional Supplementary Files [file 42003_2022_4224_MOESM3_ESM.pdf]

# Description of Additional Supplementary Files

File name: Supplementary Data

Description: The source data behind the graphs in the paper.
